# Zymophagy: Selective Autophagy of Secretory Granules

**DOI:** 10.1155/2012/396705

**Published:** 2012-04-10

**Authors:** Maria I. Vaccaro

**Affiliations:** Department of Pathophysiology, School of Pharmacy and Biochemistry, University of Buenos Aires, 956 Junin, Buenos Aires C1113AAD, Argentina

## Abstract

Timing is everything. That's especially true when it comes to the activation of enzymes created by the pancreas to break down food. Pancreatic enzymes are packed in secretory granules as precursor molecules called zymogens. In physiological conditions, those zymogens are activated only when they reach the gut, where they get to work releasing and distributing nutrients that we need to survive. If this process fails and the enzymes are prematurely activated within the pancreatic cell, before they are released from the gland, they break down the pancreas itself causing acute pancreatitis. This is a painful disease that ranges from a mild and autolimited process to a severe and lethal condition. Recently, we demonstrated that the pancreatic acinar cell is able to switch on a refined mechanism that could explain the autolimited form of the disease. This is a novel selective form of autophagy named zymophagy, a cellular process to specifically detect and degrade secretory granules containing activated enzymes before they can digest the organ. In this work, we revise the molecules and mechanisms that mediate zymophagy, a selective autophagy of secretory granules.

## 1. Introduction

Autophagy is an evolutionarily preserved cellular process that is responsible for the degradation of long-lived proteins and entire organelles to maintain intracellular homeostasis and to contribute to starvation and stress responses. Macroautophagy involves the formation of double-membrane autophagosomes around cargoes, including larger structures such as organelles and protein aggregates. Autophagosomes then fuse with lysosomes, where the degradation of the cargoes takes place. Both nonselective *bulk *autophagy and selective autophagy of specific proteins and organelles have been described [1]. Genetic analyses in yeast identified more than 30 conserved components that are required for different steps of autophagy (termed *Atg *genes) [2]. Recently, several lines of evidence suggest the existence of selective autophagic degradation pathways in physiology and disease, named, selective autophagy [3]. During selective autophagy, single cellular structures, such as protein aggregates and mitochondria are specifically sequestered by autophagosomes. 

There is emerging evidence suggesting the involvement of ubiquitin in several forms of selective autophagy process. For example, aggregate clearance by autophagy requires ubiquitylation and ubiquitin-binding receptors such as p62 (also known as SQSTM1) [4]. Ubiquitylated artificial substrates are recognized by the autophagy machinery and are specifically degraded in lysosomes by a p62-dependent mechanism [5]. Moreover, the selective degradation of excess ribosomes during starvation depends on the deubiquitylation activity of Ubp3/Bre5 [6]. The repertoire of proteins that participate in these ubiquitin-mediated pathways during different types of autophagy is an area of intensive investigation, particularly the elucidation of the role of this specific cellular program in pathophysiological processes and complex diseases such as pancreatitis. 

Zymophagy is a novel selective form of autophagy that works as a protective cell response to disease [7]. This new selective autophagic pathway is activated in pancreatic acinar cells during pancreatitis-induced vesicular transport alteration, in order to sequester and degrade potentially deleterious activated zymogen granules. In this work, we revise the molecules and mechanisms that mediate *zymophagy*, a self-eating event that protects the pancreas from self-digestion.

## 2. The Disease

### 2.1. Acute Pancreatitis

Acute pancreatitis, defined as the pancreas self-digestion, is the most frequent disease of the pancreas. During pancreatitis, ultrastructural alterations of zymogen granules are produced in a yet undefined way. These alterations are characterized by premature activation of trypsinogen to trypsin within pancreatic acinar cells leading to the progression of the disease. Most of the cases are mild acute pancreatitis resulting in a self-limiting disease, but up to 25% of the patients suffers a severe attack and around 30% of these will die [8]. The pathophysiology involves a complex cascade of events initializing in pancreatic acinar cells. An unknown trigger within the pancreas leads to conversion of digestive proenzymes (zymogens) into their active form (digestive enzymes), initiating autodigestion of the gland causing hemorrhage, necrosis, edema, and complete destruction of pancreatic parenchyma [9]. Chiari [10], more than a century ago, proposed that autodigestion by prematurely activated digestive enzymes is responsible for the onset of this debilitating disease. Although significant research effort has been expended on mechanisms responsible for this premature zymogen activation, many aspects of the pathogenesis of acute pancreatitis remain enigmatic (see [11] for a comprehensive review). The presence of autophagy has been described in dying acinar cells from human pancreatitis. Helin et al. in 1980 described the ultrastructural alterations in pancreatic acini from patients operated for acute necrotizing pancreatitis. They studied by electronic microscopy those areas of pancreatic parenchyma that show edematous inflammation under light microscopy. Their findings in acinar cells included changes in zymogen granules and an increased autophagocytosis indicated by several autophagic vesicles [12].

Cholecystokinin (CCK) is a pancreatic secretagogue that interacts with Gq-coupled receptors in the acinar cell to induce pancreatic secretion in physiological conditions. On the other hand, the hyperstimulation of CCK receptors (CCK-R) with the analogue cerulein that modifies vesicular transport leads to intracellular proteolytic enzyme activation and ultimately cell death [13]. These cellular events are characteristic of human acute pancreatitis. Moreover, autophagic morphological features were also described in cerulein-induced pancreatitis [14, 15]. Further, the secretagogue-induced model is the most commonly employed and best characterized experimental model of acute pancreatitis [16]. Interestingly, early during this experimental model, CCK-R hyperstimulation activates the selective autophagic degradation of secretory granules in the pancreatic acinar cell [7, 17].

## 3. The Cells

### 3.1. The Pancreatic Acinar Cells

The acinar cell of the exocrine pancreas produces and secretes a wide variety of potent proteolytic enzymes essential for intestinal digestion of nutrient proteins. However, these digestive enzymes are potentially harmful. Therefore, these proteases are produced as precursors (zymogens) within pancreatic acinar cells and are only activated in the duodenum. The key step in this activation process is the conversion of inactive trypsinogen to active trypsin by limited proteolysis by enteropeptidase, a highly selective trypsinogen-cleaving protease located at the luminal site of duodenal cells. The active trypsin initiates an activation cascade of digestive enzymes within the duodenum, thereby ensuring the high proteolytic capacity needed for food digestion. Under pathological conditions that cause pancreatitis, digestive zymogens undergo premature activation within the pancreatic acinar cell. High levels of activation overcome pancreas protective mechanisms, causing cell injury and acute pancreatitis [16]. However, most of the cases of acute pancreatitis are mild and autolimited. Therefore, the acinar cell may respond to the pathogenic event activating phenotypically changes that may start defense mechanisms and explain the autolimited form of the disease [17, 18].

## 4. The Organelles

### 4.1. Pathologically Activated Zymogen Granules

The pancreatic acinar cell is a highly polarized, differentiated cell whose primary function is the synthesis and secretion of digestive enzymes into the pancreatic juice. Pancreatic digestive enzymes are produced as inactive enzymes (zymogens) and stored in subcellular structures called zymogen granules, until exocytosis [16]. Zymogen granules are potentially harmful secretory granules because once activated, they release the digestive enzymes within the cell causing cell death. Thus, they are able to hydrolyze tissue parenchyma and eventually trigger a severe disease. The exact mechanisms of zymogen activation and the complete characterization of the activation compartments remain unclear [19]. However, these pathologically altered organelles containing activated zymogens may be recognized and degraded by a selective autophagic pathway that we named zymophagy [7].

## 5. The Molecules

### 5.1. VMP1

In the search for new molecules that are differentially expressed during acute pancreatitis we found a transmembrane protein that we named Vacuole Membrane Protein 1 (VMP1) [20]. The *in vivo* gen expression of VMP1 in pancreas with pancreatitis correlates with morphological features resembling autophagy [21]. VMP1 is a transmembrane protein highly activated in acinar cells early during pancreatitis-induced autophagy, and it remains in the autophagosomal membrane. We have shown that VMP1 expression is able to trigger autophagy in mammalian cells, even under nutrient-replete conditions [17]. Most of the autophagy-related proteins were described in yeast or have a yeast homologue. VMP1 does not have any known homologue in yeast, but its expression is required to start the autophagic process in mammalian cells [22]. VMP1 overexpression in mammalian cells induces the formation of numerous vesicles with ultrastructure of autophagosomes and they immunostained with LC3, the widely used marker of autophagosomes. Moreover, VMP1 expression promotes the conversion of LC3-I to LC3-II. VMP1 expression in several mammalian cell lines induces recruitment of LC3 in punctate structures, and that recruitment is inhibited when VMP1-expressing cells are treated with the autophagy inhibitor 3-methyladenine (3-MA). VMP1 endogenous expression is induced by autophagy stimuli, and its expression is required for autophagosome development. VMP1 interacts with Beclin 1 through its hydrophilic C-terminal region, the Atg domain that is essential for autophagy [17]. Recently, Tian et al. identified EPG-3/VMP1 as one of three essential autophagy genes conserved from worms to mammals, which regulates early steps of the autophagic pathway in *C. elegans* [23]. Hierarchical analyses in mammalian cells by Itakura and Mizushima [24] show that VMP1 along with ULK1 and Atg14 localize in the ER-associated autophagosome formation sites in a PI3-kinase activity-independent manner, confirming the key role of VMP1 in the formation of autophagosomes. VMP1 expression is induced by extracellular stimuli of autophagy. For instance, rapamycin, the pharmacological inhibitor of mTOR and a well-established inductor of autophagy, is able to activate VMP1 expression, and VMP1-immunostained vesicles appear in rapamycin-treated cells [17]. Other extracellular stimuli such as gemcitabine in pancreatic cancer cells [25] and streptozotocine in pancreatic beta cells [26] induce VMP1 expression and VMP1-mediated autophagy. VMP1 autophagic vesicles are present in the pancreas of rats submitted to experimental pancreatitis, showing that VMP1 is involved in the induction of autophagy during the disease. Considering that autophagy is implicated in several pathological mechanisms operating in human diseases, the activation of the VMP1 pathway may regulate potential pathophysiological processes involved in the cell response to disease. We developed the ElaI-VMP1 mouse in which acinar cell-specific constitutive expression of a VMP1-EGFP chimera induces the formation of autophagosomes within the acinar cell [17]. Thus, the ability of VMP1 expression to induce autophagy is validated through the development of this pancreas-specific transgenic mouse. In the adult normal pancreas, autophagy and VMP1 are not detected; in contrast, in VMP1-transgenic mice, multiple VMP1 and LC3 coimmunostained autophagic structures are present in pancreas cells. We use this unique tool to investigate the VMP1 pathway in autophagy during acute pancreatitis [7, 17].

### 5.2. p62/SQSTM1

Dictyostelium cells lacking Vmp1 gene show accumulation of huge ubiquitin-positive protein aggregates containing the autophagy marker Atg8/LC3 and p62 homologue [27]. The polyubiquitin-binding protein p62/SQSTM1 is degraded by autophagy. It is found in cellular inclusion bodies together with polyubiquitinated proteins and in cytosolic protein aggregates that accumulate in various chronic, toxic, and degenerative diseases. It has been shown a direct interaction between p62 and the autophagic effector proteins LC3A and -B and the related *γ*-aminobutyrate receptor-associated protein and *γ*-aminobutyrate receptor-associated-like proteins. The binding is mediated by a 22-residue sequence of p62 containing an evolutionarily conserved motif. The specific interaction between p62 and LC3 is instrumental in mediating autophagic degradation of the p62-positive bodies and p62 is required both for the formation and the degradation of polyubiquitin-containing bodies by autophagy [4].

### 5.3. USP9x

**Figure 1:**
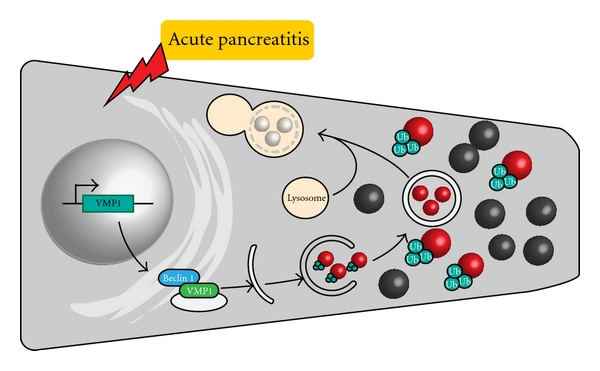
Upon acute pancreatitis, the acinar cell activates Vmp1 gene expression and triggers the autophagic degradation of those zymogen granules activated during the disease (in red) avowing the intracellular spreading of activated digestive enzymes. Altered zymogen granules are recognized by ubiquitination. Zymophagy is a novel inducible and selective form of autophagy, mediated by VMP1, which functionally links the autophagy pathway with the ubiquitin machinery to trigger a protective response to disease.

Ubiquitination is a covalent posttranslational modification of cellular proteins involving a complex enzymatic cascade. Emerging evidence suggests that many enzymes of the ubiquitination cascade are differentially expressed or activated in several diseases, including cancer and may therefore be appropriate therapeutic targets. Protein ubiquitination is a dynamic two-way process that can be reversed or regulated by deubiquitinating enzymes (DUB). The human genome codes for hundred proteins with putative DUB activity [28], which can be broadly divided into two subgroups: ubiquitin COOH-terminal hydrolase (UCH) and the ubiquitin-specific proteases (USP) [29]. USPs comprise the largest subclass of DUBs in humans, whereas only four known UCH DUBs have been described [30]. The USP9x gene is a member of the peptidase C19 family and encodes for an ubiquitin-specific protease. Ubiquitinating and deubiquitinating enzymes have emerged as key players in the regulation of membrane trafficking in organisms ranging from yeasts to mammals [31] (Figure 1).

## 6. The Selective Autophagic Pathway

During acute pancreatitis, the acinar cell activates VMP1-mediated autophagy. The immunomagnetic isolation of VMP1-autophagosomes containing zymogen granules from the EGFP-VMP1 transgenic mouse pancreas with acute pancreatitis allows the discovery of a new type of selective autophagy named zymophagy, which functions as an inducible cellular process that recognizes and degrades activated zymogen granules [7].

Zymophagy is characterized by the formation of autophagosomes containing zymogen granules. These organelles mediate the sequestration and degradation of pancreatitis-activated zymogen granules and are induced by secretagogues and probably other stimuli in acinar cells. Electron microscopy and immunofluorescence assays in human, mouse, and cultured pancreatic acinar cells show autophagic organelles at different maturation levels, suggesting that the autophagic flow progresses to acquire autolysosomal features and degrades the disease-altered secretory granules. CCK-R hyperstimulation in wild-type animals induced a markedly altered distribution pattern of the secretory granules such as fusion among zymogen granules as well as their fusion with condensing vacuoles. In addition, acinar cells lose their polarity, which results in the relocation of zymogen granules to the basolateral membrane. All these alterations in vesicular traffic are known to occur in acinar cells during acute pancreatitis and upon hyperstimulation of their CCK-R with cerulein. Surprisingly, ElaI-VMP1 mice subjected to CCK-R hyperstimulation reveal that acinar cells preserve their structure and polarity with negligible or no alteration in vesicular transport. Instead, pancreases from cerulein-treated ElaI-VMP1 mice present autophagosomes containing zymogen granules displaying a distinct localization to the apical area of the acinar cell. These autophagosomes, containing secretory granules, were easily identifiable in apical regions as round high-density structures within double-membrane vesicles. After a systematic observation, we did not find evidence of other subcellular structures, such as ribosomes or mitochondria, within these autophagosomes. The observation of different maturation levels of selective autophagic vesicles as well as the degradation of p62 provides evidence that autophagic flow remains primarily unchanged under CCK-R hyperstimulation. Interestingly, similar to the case of *in vivo* autophagosomes, *in vitro* CCK-R hyperstimulation of ElaI-VMP1 mouse isolated acini induces a subcellular change in VMP1-containing autophagosomes to the zymogen granule-rich area (apical pole), therefore, in acinar cells from the ElaI-VMP1 mice, CCK-R hyperstimulation induces a progressive flow of autophagic vesicles containing zymogen granules that accumulate at the apical pole of acinar cells [7].

The distinctive characteristic of VMP1 as an autophagosome transmembrane protein allows the isolation of autophagosomes from pancreas tissue of treated and untreated ElaI-VMP1 mice. Autophagosomes containing zymogen granules are magnetically immunopurified from the pancreas of ElaI-VMP1 mice treated with cerulein, whereas in untreated animals, either empty or cytoplasm-containing autophagosomes are purified. In untreated animals, less than 20% of the autophagosomes contains zymogen granules, whereas in the cerulein treated ones, this percentage increases up to 70%. LC3-II is present in autophagosomal fractions from both cerulein-treated and untreated specimens. Notably, strong signals of p62 and trypsinogen are found only in magnetically immunopurified autophagosomes from cerulein-treated ElaI-VMP1 mice. This finding suggests that p62, which is an ubiquitin-binding protein that interacts with LC3 [4], may function as a cargo receptor during the selective autophagic pathway. Therefore, a nonselective autophagy pathway, which involves LC3-II but not p62, is triggered by VMP1 expression in untreated mouse pancreases. On the other hand, upon CCK-R hyperstimulation, p62 is involved in VMP1-mediated selective autophagy of zymogen granules. The description of zymophagy positions VMP1 and p62 in the same selective autophagy pathway. Immunofluorescence assays and western blot analyses of proteins from isolated selective autophagosomes demonstrate that these proteins colocalize and coexist in this autophagic organelle suggesting that p62 may act as cargo receptor during VMP1-selective autophagic pathway [7].

Zymophagy selectively degrades activated zymogen granules. The intracellular activation of trypsinogen induced by hyperstimulation of CCK-R in isolated murine pancreatic acini can be detected using rhodamine 110 bis-(CBZ-L-isoleucyl-L-prolyl-L-arginine amide) dihydrochloride (BZiPAR), a cell permeable substrate that becomes fluorescent after the cleavage by the protease [32]. Upon CCK-R hyperstimulation, acinar cells from wild-type mice show early cytoplasmic trypsinogen activation, which is a hallmark of pancreatitis pathophysiology. Surprisingly, in acinar cells from ElaI-VMP1 mice, CCK hyperstimulation causes almost no activation of trypsinogen. Microscopic examinations reveal only few activated granules that highly colocalize with the VMP1-EGFP fluorescent signal showing that zymophagy selectively sequester the activated zymogen granules. Zymogen activation is an enzymatic chain reaction where initial zymogen granule alterations trigger rapid spread of active trypsin within the acinar cell. We think that the degradation of early-activated zymogen granules by zymophagy prevents this deleterious event. Interestingly, the inhibition of autophagic flow markedly increased trypsin activity within acinar cells in ElaI-VMP1 mouse pancreases under CCK-R hyperstimulation, confirming that zymophagy specifically degrades those zymogen granules that are initially activated by acute pancreatitis. This function of zymophagy is evident in the *in vivo* animal model of acute pancreatitis, where the hability of the ElaI-VMP1 mouse developing zymophagy clearly prevents the increment of enzymatic markers of pancreatic damage and pancreas morphological changes characteristic of acute pancreatitis.

Zymophagy specifically recognizes the pathologically activated zymogen granules. During zymophagy, the ubiquitin system serves as a targeting signal for zymogen granules. Proteins involved in the recognition of target for autophagy are ubiquitin-binding proteins, such as p62 that binds directly to ubiquitin and LC3. Magnetically purified VMP1-mediated autophagosomes upon CCK-R hyperstimulation contain p62 and LC3 proteins and high colocalization of ubiquitin with amylase and with trypsinogen occurs early after CCK-R hyperstimulation. Moreover, the recruitment of ubiquitin with LC3, and the ubiquitin signal within LC3 decorated vesicles along with the colocalization between VMP1 and ubiquitinated granules, and the engulfment of ubiquitinated granules by VMP1-vesicles, demonstrate the selective sequestering of ubiquitinated zymogen granules by zymophagy [7]. More important, the selectively sequestered ubiquitinated granules are those activated by CCK-R hyperstimulation. GFP-Ub-transfected acinar cells subjected to CCK-R hyperstimulation show colocalization between activated granules and ubiquitin aggregates but do not show colocalization with unaffected or normal zymogen granules, indicating that the ubiquitin system serves as a targeting signal for activated zymogen granules during zymophagy. Therefore, activated zymogen granules are directly or indirectly ubiquitinated for their recognition by autophagic membranes, in which ubiquitin acts as a label for selective engulfment. This label might be subsequently removed for completing the formation of the autophagosome or even before this engulfment step. Nevertheless, activated zymogen granules are ubiquitinated upon acute pancreatitis and the VMP1-mediated selective autophagic pathway sequesters these ubiquitinated granules [7].

Notably, the ubiquitin-specific protease USP9x is showed to be an essential component of the machinery required to selectively degraded zymogen granules during zymophagy [7]. USP9x expression is highly induced in pancreatic acinar cells under CCK-R hyperstimulation. Also, USP9x is required to promote the selective degradation of altered zymogen granules. Interestingly, VMP1 interacts with USP9x early during zymophagy, supporting a direct functional role for this ubiquitin-specific protease in this selective autophagic pathway. Furthermore, downregulation of USP9x abolished activated zymogen granule degradation in acinar cells under CCK-R hyperstimulation, confirming the essential role of VMP1-USP9x interaction for zymophagy. USP9x is likely to modulate zymogen granule selective engulfment during acute pancreatitis by modulating VMP1 or providing a preferential recognition signal for altered zymogen granules. This data demonstrates for the first time that ubiquitin modifications may possess an additional function in acinar cells by promoting the degradation of highly harmful activated zymogen granules and strongly support the idea that there is a close cooperation between the autophagy pathway and the ubiquitin machinery required for selective autophagy. Alternatively, both ubiquitination and deubiquitination of distinct critical molecules might be required for selective autophagy. Thus, due to the potential importance of this type of regulation, these findings may fuel future investigations aimed to identify the potential E3 ligases and ubiquitinated substrate(s) required for zymophagy.

Zymophagy prevents pancreatic acinar cell death induced by CCK-R hyperstimulation [7]. Autophagosome formation inhibition with 3-methyl adenine as well as autophagy flux interruption with vinblastine significantly reduces acinar cell survival in a cell model of acute pancreatitis. Moreover, VMP1 downregulation (shVMP1) also significantly decreased acinar cell survival under CCK hyperstimulation showing that VMP1 expression is required to prevent acinar cell death in acute pancreatitis. These results indicate that zymophagy prevents pancreatic cell death induced by the activation of zymogen granules [33] and confirm that endogenous VMP1 expression is activated in acinar cells to mediate zymophagy as a protective cellular response against cell death.

Furthermore, VMP1 expression and zymophagy are present in human pancreas affected by acute pancreatitis [7]. VMP1 is not detectable in human normal pancreas tissue, but its expression is activated in human pancreatitis pancreas specimens and highly colocalizes with LC3 in autophagosomes. Moreover, autophagosomes markedly colocalize with zymogen granules. Remarkable, the finding of large autolysosomes without trypsin signal in human pancreatitis pancreas supports the experimental data and suggests that affected zymogen granules are eventually degraded by zymophagy during human pancreatitis. Results collectively demonstrate a previously unrecognized function for VMP1, mediating zymophagy, a novel selective form of autophagy, which functionally links the autophagy pathway with the ubiquitin machinery to trigger a protective cell response to disease.

## 7. The Function

The description of zymophagy provides further understanding of the autophagy molecular mechanisms relevant to human diseases, particularly acute pancreatitis. This condition has classically been considered an autodigestive disorder where the inappropriate activation of trypsinogen within the pancreatic acinar cell leads to the progression of the disease. The exact mechanism for the initiation of zymogen granule activation remains a subject of intensive investigation and debate [11]. Similarly, the role of autophagy in pancreatitis is seemingly incomplete, sometimes contradictory and requires further investigations. For instance, Hashimoto et al. [34] propose that autophagy is responsible for the zymogen activation within the acinar cell in acute pancreatitis. This suggestion is mainly based on the finding that deletion of the Atg5 gene in mouse pancreatic cells seems to prevent morphological alterations induced by CCK-R hyperstimulation. However, cells lacking Atg5 and Atg7 can still perform autophagy-mediated protein degradation [35], showing that deletion of these genes does not completely abolish autophagy. Moreover, no selective autophagic features were reported in the LC3 transgenic mouse pancreas after 48 hours starvation [36]. However, the induction of autophagy by starvation did not promote any morphological evidence of acute pancreatitis [36]. Interestingly, VMP1 constitutive expression induces the formation of autophagosomes in acinar cells but does not trigger pancreatitis [7, 17]. On the other hand, Mareninova et al. [37] have proposed that lysosomal proteases cathepsin L and B are reduced, suggesting a defect in the autophagic flow during acute pancreatitis. These data also introduce the question whether the role of different types of autophagy has an impact on pancreatitis. In this regard, zymophagy is an inducible form of selective autophagy activated in response to disease. Zymophagy is not induced by starvation; it is triggered by CCK-R hyperstimulation and mediated by VMP1-USP9x-p62 autophagy-ubiquitin pathway [17]. This selective form of autophagy sequesters and degrades activated zymogen granules and prevents acinar cell death. Therefore, the outcome of pancreatitis differs when different types of autophagy pathways are considered, including selective and nonselective ones.

## 8. The Meaning

For the first time, it was demonstrated that there is activation of the VMP1-autophagic pathway and a selective autophagy of secretory granules during human pancreatitis [7]. These findings lead us to discuss how this knowledge fits the current theoretical framework regarding the occurrence and progression of this disease. Acute pancreatitis is a frequent, painful, and often deadly disease that ranges from a mild, edematous, and autolimited process, to a severe necrotizing and eventually lethal condition. Additionally, the etiology of this disease is diverse, and different stimuli can initiate the same autodigestion cascade via different mechanisms. VMP1 expression is activated during acute pancreatitis in humans, and there is formation of autophagosomes where p62, LC3, and zymogen granules colocalize [7]. The selective autophagic degradation of zymogen granules (Zymophagy) is a protective cell response that could explain, at least in part, the autolimited form of this disease seen in many patients. Hence, it is tempting to speculate that the more efficient zymophagic response by the pancreatic acinar cell, the less severity of the disease. In contrast, severe forms of pancreatitis offering an excess of cargo and an accelerated rate of degradation might overcome or disrupt the protective capacity of this selective autophagic process. The latter possibility is suggested by our studies since the inhibition of the autophagic flow impairs the protective role of zymophagy [17]. Interestingly, Fortunato and Kroemer [38] findings, regarding the reduction of the autophagosomelysosome fusion in human alcoholic pancreatitis, further support our hypothesis.

Finally, this novel autophagic pathway that selectively degrades altered secretory granules might be involved in other pathological processes affecting secretory cells, such as pancreatic beta cells in diabetes mellitus [26] or Paneth cells in Chron's disease [39]. Therefore, more studies on selective autophagy as a programmed cell response to pathological processes that affect protein secretion are important to significantly expand our knowledge of the role of autophagy in both cell biology and human disease.

## 9. Conclusions

Zymophagy revels a critical function of autophagy in secretory granule homeostasis and cell response to injury. It consists in the selective degradation of disease-induced activated secretory granules. This process can be reconstructed by the hyperstimulation of Gq-coupled receptors with CCK in a transgenic mouse model for studying VMP1-induced autophagy in pancreatic acinar cells (ElaI-VMP1 mice). A VMP1-USP9x-p62 molecular pathway is involved in this selective autophagic process. Zymophagy degrades the activated granules avoiding the spreading of their contents into the cytoplasm, thus preventing further trypsinogen activation and cell death.
